# Primary care physicians’ knowledge, attitudes and concerns about bariatric surgery and the association with referral patterns: a Swedish survey study

**DOI:** 10.1186/s12902-021-00723-8

**Published:** 2021-04-08

**Authors:** Ensieh Memarian, Daniel Carrasco, Hans Thulesius, Susanna Calling

**Affiliations:** 1 Department of Clinical Sciences in Malmö, Lund University, Internal Medicine Research Group, Jan Waldenströms gata 15, 5th floor, Skane University Hospital, S-20502 Malmö, Sweden; 2 Center for Primary Health Care Research Region Skåne and Department of Clinical Sciences in Malmö, Lund University, Lund, Sweden; 3 Linnaeus University, Kalmar, Sweden

**Keywords:** Obesity, Bariatric surgery, Survey study, Primary care physician, Knowledge, Referral, Attitudes

## Abstract

**Background:**

Obesity prevalence is increasing globally. Bariatric surgery is an effective treatment for severe and complex obesity resulting in significant and sustained weight loss. In Sweden, most bariatric surgery patients are referred by primary care physicians. We aimed to explore barriers for physicians to refer patients with severe and complex obesity for bariatric surgery.

**Methods:**

A questionnaire survey was in 2019 emailed to 1100 primary care physicians in the Skåne and Kronoberg regions in south Sweden. The survey focused on referral patterns, knowledge and attitudes towards bariatric surgery and concerns about postoperative complications. We created different statistical indices for referral patterns, knowledge, attitudes and concerns about bariatric surgery. To analyze the correlation between these indices, we did Spearman’s correlations and regression analyses.

**Results:**

Of 1100 email respondents, we received 157 (14%) completed surveys. Among 157 physician respondents, 73% answered that they had good knowledge about the referral criteria for bariatric surgery, whereas 55 and 60% answered correctly to two items on criteria for bariatric surgery. A majority of respondents (84%) stated that their patients initiated referral to bariatric surgery. Half of the respondents had concerns about postoperative medical and surgical complications, but another half had a positive attitude to bariatric surgery as a treatment for obesity comorbidities. Almost half of the respondents (44%) answered that they needed to learn more about bariatric surgery.

We found significant positive correlations between high knowledge and referral patterns (r = 0.292, *p* < 0.001) and positive attitudes (r = 0.235, *p* < 0.001) respectively. We found significant reverse correlations between concerns and referral patterns (r = − 0.355, *p* < 0.001) and between positive attitudes and concerns (r = − 0.294, p < 0.001). In logistic regression high levels of concerns explained low willingness to refer for bariatric surgery (Odds Ratio 0.2, 95% confidence interval 0.1–0.7).

**Conclusion:**

According to this Swedish survey among primary care physicians, high levels of concerns about bariatric surgery among physicians seemed to be a barrier to refer patients with severe and complex obesity for bariatric surgery. Since high knowledge about obesity and bariatric surgery correlated negatively to concerns and positively to favorable attitudes to bariatric surgery, more knowledge about obesity and bariatric surgery is warranted.

**Supplementary Information:**

The online version contains supplementary material available at 10.1186/s12902-021-00723-8.

## Background

The prevalence of obesity is increasing globally, and half of the population aged 16–84 years in Sweden was overweight or obese in 2018 [1]. Body mass index (BMI) is used to define overweight (BMI 25–29.99 kg/m^2^) and obesity (BMI ≥ 30 kg/m^2^) in adults. According to WHO, BMI ≥ 40 kg/m^2^ is classified as obesity class III, in this article defined as severe and complex obesity.

An increased BMI is a risk factor for several comorbidities such as diabetes type 2, obstructive sleep apnea, cardiovascular diseases, infertility in women, several types of cancer and depression [2–7]. Obesity is strongly associated with higher mortality, decreased quality of life and higher costs for the health care system and society [7] due to higher rates of absenteeism, productivity loss and premature mortality [8].

Bariatric surgery has shown positive effects on morbidity and mortality in individuals with severe and complex obesity and is an effective treatment resulting in significant and sustained weight loss [9–11]. Bariatric surgery is also considered cost effective for society due to decreased costs related to obesity-related comorbidities [9, 12, 13].

According to Swedish national guidelines, individuals who do not lose weight by nonsurgical obesity treatment and have a BMI ≥ 40 kg/m^2^ or a BMI ≥ 35 kg/m^2^ in addition to a major obesity-related comorbidity, are eligible for bariatric surgery paid for by public health care [14].

Although severe and complex obesity is equally common among men and women in Sweden [14], the majority (75%) of those who undergo bariatric surgery in Sweden are women [14–17]. According to a global report from 51 countries the proportion of female patients undergoing bariatric surgery 2014–2018 was 73.7% [18]. This indicates that many men who are eligible for bariatric surgery do not receive this treatment.

In 2007 an obesity task force was created on behalf of the Swedish National Board of Health, the Swedish Municipalities & Counties and the Swedish Medical Association [14]. The obesity task force estimated that there was a medical indication for between 10,000 and 15,000 obesity surgeries annually. In 2006, approximately 1500 operations were performed and in 2008 almost 3300 bariatric procedures were performed in Sweden, [14]. Since 2012, approximately 7000 surgeries were performed annually [19]. This indicates that the number of bariatric procedures is considerably lower than the potential need based on present indications. In the USA only < 1% of US adults with severe and complex obesity undergo bariatric surgery annually [20].

Considering the relatively small proportion of eligible patients that undergo bariatric surgery, it is important to understand barriers to the treatment. In a systematic review it was seen that both patients and referring physicians had considerable concerns about the outcomes and safety of bariatric surgery, and the physicians acknowledged that they had limited knowledge about obesity treatment alternatives [21]. A Danish survey, showed that Danish primary care physicians had a high degree of reluctance towards bariatric surgery as a treatment option for obesity due to fear of postoperative medical and surgical complications [22].

In Sweden, the majority of patients are referred to bariatric surgery by primary care physicians. The results of a survey from USA showed that the probability that primary care patients would seriously consider bariatric surgery was five times higher if their physicians recommended bariatric surgery [23]. However only 20% of the patients in that study reported that they were recommended bariatric surgery by their primary care physician.

Our primary aim of the current study was to assess referral patterns, knowledge, attitudes and concerns about bariatric surgery for patients with obesity among primary care physicians through an electronic questionnaire. Our secondary aim was to explore potential barriers for referral of patients to bariatric surgery by investigating possible correlations between the physicians’ knowledge, attitudes and concerns about bariatric surgery, and if these factors might influence the suggested referral patterns. Our hypothesis was that low knowledge about obesity and bariatric surgery in primary care physicians might correlate to negative attitudes or concerns, which consequently would be associated with less referral for bariatric surgery for patients with severe and complex obesity.

## Methods

An electronic questionnaire survey was in 2019 emailed to all primary care physicians with potentially available email addresses in Skåne and Kronoberg regions in south Sweden. We chose Skåne and Kronoberg regions since the authors worked in these two regions and had access to primary care physicians’ email addresses. Skåne is Sweden’s third most populated region and Kronoberg represents a 2% cross section of Sweden both regarding population demographics and surface area. We had access to the email addresses through the regional councils.

The potential participants were informed briefly about obesity, the purpose of the study and that their participation was voluntary and that responses were collected anonymously and could not be traced back to any of the participants. They were also informed that they could terminate their participation at any time. A survey response by the physicians was considered as a consent to participate. According to Swedish legislation, application to the Ethical Review Authority (EPM) was not required, as the survey was anonymous and did not contain what the EPM considers as sensitive data.

We designed the electronic questionnaire by taking the following steps:

- We, the authors, discussed barriers affecting physicians’ decision making to refer potential patients to bariatric surgery. The discussion was based on our own experiences and those from other primary care colleagues.
- We searched the literature for other questionnaire surveys that had one or more items of interest for our study and found four studies [22, 24–26]. Items of interest were chosen, and additional items were created. The items focused on referral patterns (e. g. whether it is primarily the physician or the patient who suggests referral to bariatric surgery), knowledge (e. g. BMI criteria for referral and medical effects of bariatric surgery) and attitudes towards bariatric surgery (e. g. having bad experiences about bariatric surgery and overall impressions about bariatric surgery) and concerns about postoperative complications (e. g. adverse consequences of surgery). Most of the items were graded on a 5-point Likert scale - 1 = strongly disagree; 2 = disagree; 3 = neither agree nor disagree; 4 = agree; 5 = strongly agree and 6 = do not know.
- We translated items from other questionnaire surveys from English to Swedish and from Swedish back to English consecutively by two independent researchers to ensure that the meaning of the items did not alter after the translation.
- We invited 20 primary care physicians to answer and give comments on the first version of the survey in order to assess the content and the face validity of the survey.
- We altered the survey based on the comments, which were found to be applicable to the purpose of the study.
- The final version of the questionnaire contained 59 items and was created electronically in REDCap®, a web application for handling electronic questionnaires.
- In order to check if the survey link worked properly, we sent the survey to the authors and a sample of 10 email addresses of colleagues at our clinic. This sample of 10 emaila ddresses were not included in the final mail list used for collecting the survey data.

The final version of the questionnaire was electronically sent out to all physicians, with available email addresses, working in primary care in Skåne and Kronoberg regions in south Sweden. The survey was sent out the first time in July 2019. We send out two reminders, one in August and one in October 2019. The total duration of the study was 4 months.

The questionnaire contained demographic information such as age, gender, years of experience, and medical specialization.

Data were analyzed and estimated by descriptive statistics and regression analyses. We developed indices for items regarding referral patterns (items 7–10), knowledge (items 23–41), attitudes (items 47, 51, 53) and concerns (items 42–46) about bariatric surgery. A detailed description of how the indices were developed is presented in Additional file 1: Appendix 1. A brief description of the process is as follows: Most of the items were coded in accordance with a Likert scale, except for a few items (e. g. items 9, 23, 53) that were coded reversely. For example, higher scores for items about willingness to refer patients generated a higher index score for referral patterns etc. The English version of the questionnaire is attached as Additional file 2: Appendix 2. Spearman’s correlations and regression analyses were used to assess the associations between the scores of the indices. The SPSS statistics program Data Editor, version 25, was used and *p* < 0.05 was considered statistically significant.

## Results

### Demographic data

The questionnaire survey was emailed to 1100 primary care physicians in Skåne and Kronoberg regions and 157 responded (14%). A total of 11 questionnaires were not complete and had internal missing answers for 1–4 items. Of the 157 respondents, 41% (*n* = 65) were male and 58% (*n* = 91) female (1 missing). Most of the respondents (*n* = 105, 67%) claimed to be specialists in general practice and 53 of them answered that they had worked more than ten years in primary care. Most of the respondents (*n* = 120, 77%) worked in public primary care and 23% (*n* = 36) worked in private primary care, see Table 1.

**Table 1:** Demographic data for the respondents

	Men	Women	Total
**Number of participants**	65 (41%)	91 (58%)	157 (100%)
**Gender (missing = 1)**			
**Age**			
**Age < 35 year**	7 (11%)	14 (15%)	21 (13%)
**Age 35–49 year**	23 (35%)	47 (51%)	70 (45%)
**Age > 49 year**	35 (54%)	31 (34%)	66 (42%)
**Speciality (missing = 1)**			
**Specialist in general practice**	41 (63%)	64 (70%)	105 (67%)
**Primary care physician without speciality in general medicine**	23 (35%)	28 (30%)	51 (33%)
**Speciality experience (*n*** **= 109)**			
**Worked as a specialist in general practice 0–5 year**	13 (20%)	19 (21%)	32 (20,5%)
**Worked as a specialist in general practice 6–10 year**	7 (11%)	17 (19%)	24 (15,5%)
**Worked as a specialist in general practice > 10 year**	25 (38%)	28 (32%)	53 (50%)
**Full-time/part-time (missing = 3)**			
**Working full-time**	41 (63%)	36 (40%)	77 (50%)
**Working part-time**	22 (34%)	55 (60%)	77 (50%)
**Public/private clinic (missing = 1)**			
**Public primary care**	48 (74%)	72 (79%)	120 (77%)
**Private primary care**	17 (26%)	19 (21%)	36 (23%)

### Referral pattern

**Fig. 1:**
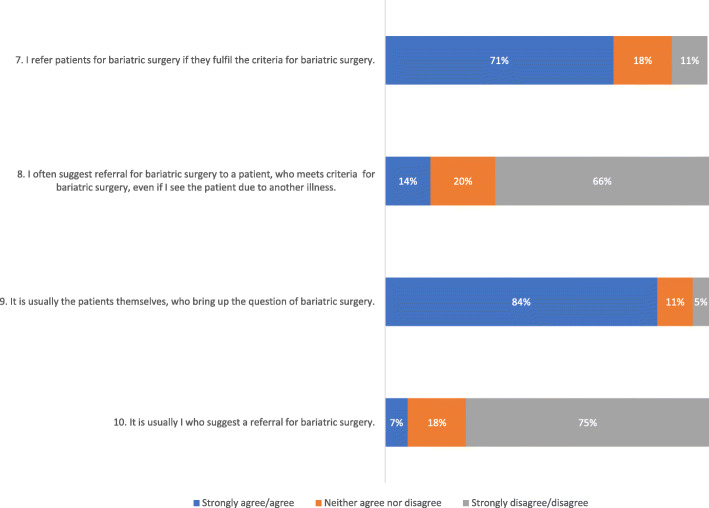
Referral patterns for bariatric surgery according to a survey to 157 Swedish primary care physicians (items 7-10). Values are displayed as percentages and numbers

Most of the physicians (*n* = 111, 71%) stated that they agreed or strongly agreed to referring patients to bariatric surgery if patients met the required national referral criteria. Only 7% (*n* = 11) of physicians actively suggested bariatric surgery to patients with obesity (item 10) whereas 84% (*n* = 132) answered that it is most often the patients that request referral to bariatric surgery (item 9), see Fig. 1.

More than half (*n* = 89, 57%) of the physicians stated that they predominantly referred women for bariatric surgery. Many physicians (*n* = 63, 40%) suggested that the gender difference was explained by women requesting referral for surgery more often than men.

More than half of the physicians (*n* = 89, 57%) met 1–2 patients per month that could be a candidate for bariatric surgery and 64% (*n* = 100) had referred 1–5 patients for bariatric surgery in the last five years. However, 11% (*n* = 17) answered that they had not referred anyone for bariatric surgery during the last five years (item 21).

More than half of the physicians (*n* = 94, 60%) had refused to refer patients to bariatric surgery despite the patient’s wish, whereas 29% (*n* = 45) had never denied a patient a referral. Most of the physicians (*n* = 94, 60%) had denied referral for bariatric surgery due to non-eligibility for bariatric surgery, 25% (*n* = 39) had denied referral due to uncertainty if the patient could follow the postoperative advices, and 19% (*n* = 30) had denied referral due to patients’ cognitive problems (item 57).

### Knowledge about obesity and bariatric surgery

A majority of respondents (73%, *n* = 115) agreed or strongly agreed to having good knowledge about the referral criteria for bariatric surgery, whereas 55% (*n* = 86) and 60% (*n* = 94) answered correctly to the two items on BMI criteria for bariatric surgery (items 35 and 36).

**Fig. 2:**
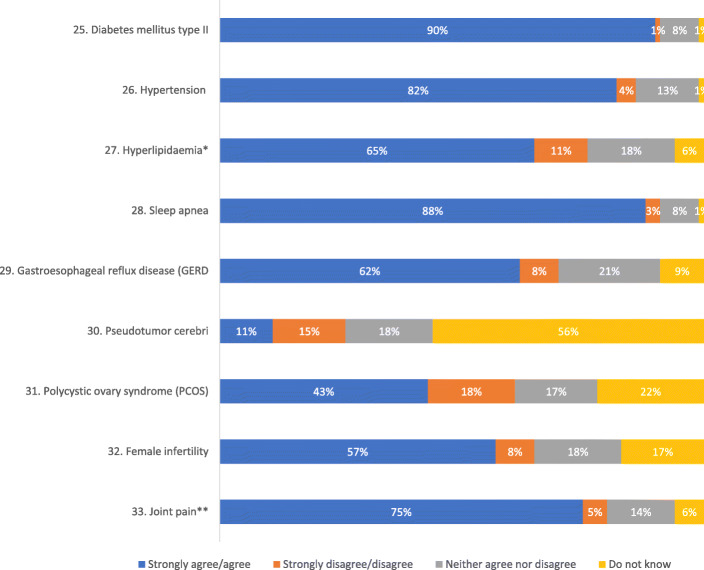
Knowledge about positive effects of bariatric surgery for nine different presented health conditions according to a survey to 157 Swedish primary care physicians (items 25-33). * = Missing =1, ** = Missing =2. Values are displayed as percentages

Most respondents (*n* = 141, 90%) agreed or strongly agreed that bariatric surgery had positive effects on type 2 diabetes, obstructive sleep apnea (*n* = 138, 88%), hypertension (*n* = 129, 82%) and joint pain (*n* = 118,75%) see Fig. 2.

While more than half of the physicians (*n* = 85, 54%) stated that diet and exercise are effective methods for sustained weight loss in patients with severe and complex obesity, only 25% (*n* = 39) stated that they agreed or strongly agreed that bariatric surgery is the only effective treatment for sustained weight loss in patients with severe and complex obesity (item 24). In response to the item about mortality associated to bariatric surgery, 57% (*n* = 89) answered correctly that perioperative mortality is < 1% (item 40).

Nearly half of the physicians (*n* = 69, 44%) indicated that they needed more education about obesity and bariatric surgery. Most of the physicians (*n* = 93, 59%) stated that they felt competent to discuss bariatric surgery as an alternative treatment for their patients with severe and complex obesity and 63% (*n* = 99) felt competent to take care of patients after bariatric surgery (items 49 and 50).

### Attitudes and concerns about bariatric surgery

Nearly half of the physicians (*n* = 75, 48%) had a positive attitude to bariatric surgery as a treatment for obesity related disease. According to 46% (*n* = 72) of the physicians the advantages of bariatric surgery outweigh the risks (item 51).

When respondents were asked if they were confident to prescribe non-surgical treatment for weight management among patients with severe and complex obesity, 41% (*n* = 64) stated that they disagreed or strongly disagreed, while 26% (*n* = 41) stated that they agreed or strongly agreed (item 48).

**Fig. 3:**
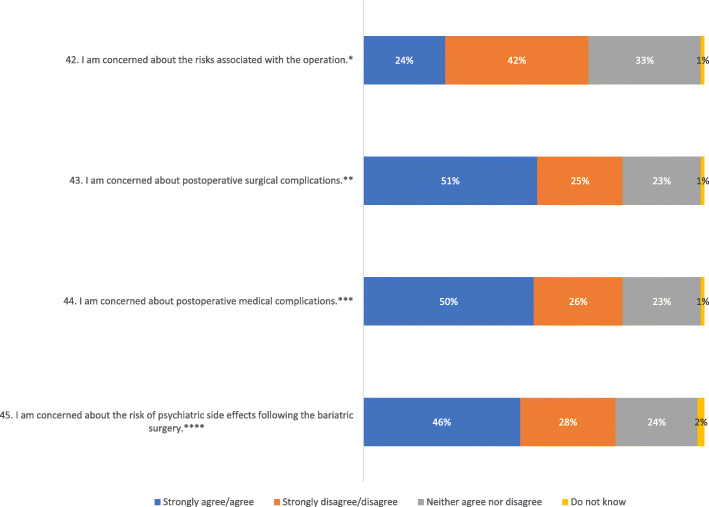
Concerns for risks associated with bariatric surgery - postoperative as well as long term risks - according to a survey to 157 Swedish primary care physicians (items 42–45). * Missing = 1, ** Missing = 1, *** Missing = 1, ****  Missing = 2. Values are displayed as percentages and numbers

Nearly half of the physicians (*n* = 66, 42%) stated that they were not concerned about the risks associated with bariatric surgery. Half of the physicians were concerned about postoperative medical (*n* = 78, 50%) and surgical complications (*n* = 79, 51%), and 46% (*n* = 71) stated that they were concerned about psychiatric side effects after bariatric surgery, see Fig. 3: The Swedish primary care physician’s concern for risks associated with bariatric surgery and postoperatively. Values are displayed as percentages and numbers * Missing = 1, ** Missing = 1, *** Missing = 1, **** Missing =2.

### Correlations and regression analysis

We created different statistical indices for referral patterns, knowledge, attitudes and concerns about bariatric surgery to be able to analyze the correlation between these items. Table 2 presents the description of these indices.

**Table 2:** Description of indices for knowledge, attitudes, referrals and concerns derived from a survey about referral patterns, knowledge and attitudes towards bariatric surgery and concerns about postoperative complications

Index	Numbers (***N***)	Mean value^*^	Median^**^	Min	Max	Standard deviation
**Knowledge**^**1**^	157	11.5	12	0	20.5	3.72
**Attitudes**^**2**^	156^**3**^	9.6	10	4	13	1.39
**Referrals**^**4**^	157	12.2	12	4	19	2.44
**Concerns**^**2**^	155^**6**^	16.4	16	6	25	4.22

** The value separating the higher half from the lower half of the data sample

^1^Item 23–41, item 41 gives maximum 3 points

^2^Item 47, 51 and 53 reverse

^3^Missing = 1

^4^Item 7, 8, 10 and 9 reverse

^5^Item 42–46

^6^Missing = 2

*The sum of the values divided by the number of values.

High knowledge was defined when the respondents answered correctly to the items regarding knowledge (items 23–41). We found significant positive correlations between high knowledge and referral patterns (r = 0.292, *p* < 0.001) and between high knowledge and positive attitudes towards bariatric surgery (r = 0.235, *p* < 0.001) i. e. high knowledge was associated with higher willingness to refer and more positive attitudes to bariatric surgery.

We found significant reverse correlations between concerns and referral patterns (r = − 0.355, *p* < 0.001) and between knowledge and concerns (r = − 0.255, *p* < 0.001), i. e. poor knowledge was associated with more concerns, and more concerns was associated with lower referral to bariatric surgery. More correlations are presented in Table 3.

**Table 3:** Spearman’s correlation analysis regarding indices for knowledge, referrals, concerns, and attitudes derived from a survey about referral patterns, knowledge and attitudes towards bariatric surgery and concerns about postoperative complications

Indices	Correlation coefficient (r)	***p***-value
**Knowledge and referrals**	0.29	< 0.05
**Referrals and concerns**	−0.36	< 0.05
**Knowledge and concerns**	−0.26	< 0.05
**Knowledge and attitudes**	0.24	< 0.05
**Referrals and attitudes**	0.28	< 0.05
**Attitudes and concerns**	−0.25	< 0.05

The correlations are is significant for *p*-values < 0,05

There was a positive correlation between physician age and referral pattern (r = 0.27, p < 0.001) and physician age and attitude (r = 0.19, *p* = 0.02). Female physician gender was associated with more concerns (r = 0.195, *p* = 0.016).

Additionally, we dichotomized the variables concerns and referral patterns and used the highest quartiles in logistic regression analyses showing that high levels of concern were associated with low willingness to refer for bariatric surgery (Odds Ratio 0.2, 95% confidence interval 0.1–0.7, *p*<0.001). Table 4 presents number of individuals with high and moderate/low concern and the willingness to refer for bariatric surgery. As presented in the table there were only 5 respondents (3%) who had high concerns and still were willing to refer to bariatric surgery.

**Table 4:** Cross table for willingness to refer for bariatric surgery with high versus low/moderate concerns. Odds Ratio 0.2, 95% confidence interval 0.1–0.7, *p*<0.001

	Low to moderate willingness to refer for bariatric surgery	High willingness to refer for bariatric surgery	Total
**Low to moderate concerns about bariatric surgery**	76	38	114
**High concerns about bariatric surgery**	38	5	43
**Total**	114	43	157

## Discussion

The principal findings of this study were that Swedish primary care physicians’ knowledge about obesity and bariatric surgery as well as their referral willingness were significantly associated with their concerns about and attitudes to bariatric surgery. The importance of treating obesity to prevent morbidity and premature mortality has been highlighted by the current COVID-19 pandemic where incremental BMI classified obesity was indeed an important risk factor for developing critical COVID-19 infection requiring invasive mechanical ventilation [27].

In our study, in the majority of cases, it was not the primary care physicians but the patients who brought up the question of referral to bariatric surgery. Similar findings were seen in a US study where primary care physicians rarely brought up the option of bariatric surgery themselves [28], and in a Danish study where the patients were the main drivers in requesting bariatric surgery [22].

Although more than half of the primary care physicians in our study reported that they met 1–2 patients per month that could be candidates for bariatric surgery, less than two third had referred 1–5 patients during the last five years, and one in nine had referred none. Furthermore, 44% of the physicians answered that they needed more education about obesity and bariatric surgery. These findings indicate that the primary care physicians lacked knowledge in this field and may need to be more active to recommend surgical options as a treatment for obesity to those patients who meet the criteria for bariatric surgery. There was a discrepancy between subjective perception about knowledge of referral criteria and the actual knowledge among respondents in our study. This further indicates the need for more training and education about obesity management for primary care physicians.

The responses also indicated a gender difference, with a majority of referrals suggested for women patients. Similar findings were reported in a systematic review from 2015 where women were more likely than men to seriously consider bariatric surgery [21].

Earlier studies have shown that lifestyle modifications such as diet and exercise most often are ineffective methods for sustained weight loss in patients with severe and complex obesity, and that the most effective weight loss programs only resulted in a sustained weight loss of 10% [23, 24]. It is not fully clear whether this small weight loss have enough impact on comorbidities in individuals with severe and complex obesity [29, 30]. Despite these findings, more than half of the respondents in our study believed that diet and exercise is an effective treatment for sustained weight loss in patients with severe and complex obesity. Also, despite that several studies indicate that the most effective method for significant and sustained weight loss is bariatric surgery, only a quarter of the primary care physicians in our study answered that bariatric surgery is the only effective treatment [9, 31]. These results emphasize the need for more education about obesity and primary care guidelines regarding obesity treatment to increase awareness among primary care physicians.

Our results show a significant negative correlation between the respondents’ concerns and referral patterns - the most prominent correlation among our analyses. Yet, a slight majority answered correctly to the mortality rate associated with bariatric surgery (mortality < 1%) [32]. High levels of concerns about postoperative surgical and medical complications might thus explain reluctancy to suggest bariatric surgery as a weight loss method in severe and complex obesity. Similar findings were reported in a Danish study [22]. A study from the USA reported that the most common reason for being uncertain about referring patients to bariatric surgery is the risk of complications and death [25]. In another recent study, the single highest reported barrier to referral for bariatric surgery was concerns of surgical complications and side effects [33]. Yet, the results of a meta-analysis showed that short-term all-cause mortality for bariatric surgery was 0.18% and for long-term mortality a reduction of 41% in all-cause mortality was seen for operated patients as compared to controls [34]. Another recent 24 yearfollow-up of the Swedish Obese Subjects Study showed that bariatric surgery was associated with longer life expectancy compared to usual obesity care, among 4047 patients with obesity [35].

The results of our study showed a significant positive correlation between high knowledge and referral and a significant negative correlation between high knowledge and concerns. This indicates that knowledge about obesity and bariatric surgery among primary care physicians is a factor that might affect referral patterns and concerns. Our data also showed that high knowledge correlated with a positive attitude. Almost half of the respondents stated that they needed more education about bariatric surgery. Two earlier studies reported that poor knowledge about bariatric surgery among primary care physicians seemed to be a barrier for bariatric surgery referral [36, 37]. Some other misconceptions about obesity among primary care physicians such as obesity “is not a disease”, obesity “is due to poor self-control”, “lifestyle change is the most effective weight loss method” and “obesity is not a priority to treat”, were also bariatric surgery barriers related to insufficient knowledge [28, 38–40].

### Limitations and strengths

One limitation of this study is the low response rate of 14% which is however similar to that of many other email surveys [41] and could partly be explained by email lists that were not updated since we received many automatic replies about invalid addresses. One might speculate that non-responders may have a different attitude and knowledge compared to responders, such as responders being more interested in obesity and bariatric surgery as a method of weight loss. However, our results are in concordance with results from a Danish primary care physician study [22]. A mixed methods study, i.e. qualitative free text data in combination with closed questions, might have given a more comprehensive understanding of knowledge and attitudes among primary care physicians. Another limitation of our study is that the survey was only sent to primary care physicians in two Swedish regions. Thus, our results may not give a fair picture of knowledge and attitudes of all Swedish primary care physicians.

One strength of this study is that it is the first Swedish survey about bariatric surgery attitudes in primary care physicians. Another strength is the analysis of correlation between knowledge, concerns, attitudes and referral patterns. The survey items were formulated both negatively and positively in order to reduce an acquiescence response set. Some of the survey items were used in other survey studies which make them comparable and they were translated from English to Swedish and back translated from Swedish to English by two independent researchers to ensure that the meaning of the items was not lost in translation. The researchers were fluent in both languages.

## Conclusion

This survey study reveals novel information about primary care physicians’ knowledge, attitudes and concerns about bariatric surgery, in two regions in the south of Sweden. The physicians’ knowledge about obesity and bariatric surgery was related to concerns and attitudes about bariatric surgery and high levels of concerns and negative attitudes seemed to be a barrier for referring patients with severe and complex obesity to bariatric surgery.

Since there is evidence showing increased life expectancy after bariatric surgery, primary care physicians need to be more aware of benefits of surgical treatment and should consider the bias that they meet more patients in daily practice with postoperative complications and unsuccessful results rather than patients who do not encounter much problems and where benefits outweighs the complications. Primary care physicians should also be more active to suggest surgery as obesity treatment for their patients, especially male patients who are not usually the main drivers in requesting bariatric surgery. More evidence-based education and training in the field of obesity and its treatment is probably warranted.

## Supplementary Information

**Additional file 1: Appendix 1.** How we developed the indices

**Additional file 2: Appendix 2.** English translation of the questionnaire

## Data Availability

The datasets used and analysed during the current study are available from the corresponding author on reasonable request.

## Abbreviations

BMI: Body mass index
EPM: Ethical Review Authority
WHO: World Health Organization

## References

[1] Folkhälsomyndigheten. Statistik övervikt och fetma. 2019, 4 November [Available from: https://www.folkhalsomyndigheten.se/livsvillkor-levnadsvanor/fysisk-aktivitet-och-matvanor/statistik-overvikt-och-fetma/.

[2] WHO. Obesity and overweight 16 Februari, 2017 [Available from: https://www.who.int/news-room/fact-sheets/detail/obesity-and-overweight.

[3] Poirier P, Giles TD, Bray GA, Hong Y, Stern JS, Pi-Sunyer FX, Eckel RH (2006). Obesity and cardiovascular disease: pathophysiology, evaluation, and effect of weight loss. Arterioscler Thromb Vasc Biol.

[4] Marchesini G, Moscatiello S, Di Domizio S, Forlani G (2008). Obesity-associated liver disease. J Clin Endocrinol Metab.

[5] Roberts RE, Deleger S, Strawbridge WJ, Kaplan GA (2003). Prospective association between obesity and depression: evidence from the Alameda County study. Int J Obes Relat Metab Disord.

[6] van Kruijsdijk RC, van der Wall E, Visseren FL (2009). Obesity and cancer: the role of dysfunctional adipose tissue. Cancer Epidemiol Biomark Prev.

[7] Long DA, Reed R, Lehman G (2006). The cost of lifestyle health risks: obesity. J Occup Environ Med.

[8] Hammond RA, Levine R (2010). The economic impact of obesity in the United States. Diab Metab Syndr Obes.

[9] Sjostrom L, Narbro K, Sjostrom CD, Karason K, Larsson B, Wedel H (2007). Effects of bariatric surgery on mortality in Swedish obese subjects. N Engl J Med.

[10] Sjostrom L (2008). Bariatric surgery and reduction in morbidity and mortality: experiences from the SOS study. Int J Obes.

[11] Rubio-Almanza M, Cámara-Gómez R, Hervás-Marín D, Ponce-Marco JL, Merino-Torres JF (2018). Cardiovascular risk reduction over time in patients with diabetes or pre-diabetes undergoing bariatric surgery: data from a single-center retrospective observational study. BMC Endocr Disord.

[12] Cremieux PY, Buchwald H, Shikora SA, Ghosh A, Yang HE, Buessing M (2008). A study on the economic impact of bariatric surgery. Am J Manag Care.

[13] Buchwald H, Avidor Y, Braunwald E, Jensen MD, Pories W, Fahrbach K, Schoelles K (2004). Bariatric surgery: a systematic review and meta-analysis. JAMA..

[14] Socialstyrelsen SKoL, Svenska Läkarsälskapet,. Nationella indikationer för obesitaskirurgi. 2007, 28 November [Available from: https://www.remittent.se/Files-sv/Videoarkiv/Bilder/Obesitas/NIOK.pdf.

[15] Memarian E, Calling S, Sundquist K, Sundquist J, Li X (2014). Sociodemographic differences and time trends of bariatric surgery in Sweden 1990-2010. Obes Surg.

[16] Memarian E, Sundquist K, Calling S, Sundquist J, Li X (2015). Country of origin and bariatric surgery in Sweden during 2001-2010. Surg Obes Relat Dis.

[17] Memarian E, Sundquist K, Calling S, Sundquist J, Li X (2019). Socioeconomic factors, body mass index and bariatric surgery: a Swedish nationwide cohort study. BMC Public Health.

[18] Welbourn R, Hollyman M, Kinsman R, Dixon J, Liem R, Ottosson J, Ramos A, Våge V, al-Sabah S, Brown W, Cohen R, Walton P, Himpens J (2019). Bariatric surgery worldwide: baseline demographic description and one-year outcomes from the fourth IFSO global registry report 2018. Obes Surg.

[19] Sundbom M, Hedberg J, Marsk R, Boman L, Bylund A, Hedenbro J, Laurenius A, Lundegårdh G, Möller P, Olbers T, Ottosson J, Näslund I, Näslund E (2017). Substantial decrease in comorbidity 5 years after gastric bypass: a population-based study from the Scandinavian obesity surgery registry. Ann Surg.

[20] Nguyen NT, Masoomi H, Magno CP, Nguyen XM, Laugenour K, Lane J (2011). Trends in use of bariatric surgery, 2003-2008. J Am Coll Surg.

[21] Funk LM, Jolles S, Fischer LE, Voils CI (2015). Patient and referring practitioner characteristics associated with the likelihood of undergoing bariatric surgery: a systematic review. JAMA Surg.

[22] Stolberg CR, Hepp N, Juhl AJA, CD B, Juhl CB (2017). Primary care physician decision making regarding referral for bariatric surgery: a national survey. Surg Obes Relat Dis.

[23] Wee CC, Huskey KW, Bolcic-Jankovic D, Colten ME, Davis RB, Hamel M (2014). Sex, race, and consideration of bariatric surgery among primary care patients with moderate to severe obesity. J Gen Intern Med.

[24] Tork S, Meister KM, Uebele AL, Hussain LR, Kelley SR, Kerlakian GM (2015). Factors Influencing Primary Care Physicians’ Referral for Bariatric Surgery. JSLS.

[25] Perlman SE, Reinhold RB, Nadzam GS (2007). How do family practitioners perceive surgery for the morbidly obese?. Surg Obes Relat Dis.

[26] Sarwer DB, Ritter S, Wadden TA, Spitzer JC, Vetter ML, Moore RH (2012). Physicians' attitudes about referring their type 2 diabetes patients for bariatric surgery. Surg Obes Relat Dis.

[27] Simonnet A, Chetboun M, Poissy J, Raverdy V, Noulette J, Duhamel A, et al. High prevalence of obesity in severe acute respiratory syndrome coronavirus-2 (SARS-CoV-2) requiring invasive mechanical ventilation. Obesity (Silver Spring). 2020. 10.1002/oby.22831 PMC7262326 32271993

[28] Funk LM, Jolles SA, Greenberg CC, Schwarze ML, Safdar N, McVay MA (2016). Primary care physician decision making regarding severe obesity treatment and bariatric surgery: a qualitative study. Surg Obes Relat Dis.

[29] Atkinson RLDW, Foreyt JP (1993). Very low-calorie diets. National Task Force on the prevention and treatment of obesity, National Institutes of Health. JAMA..

[30] Safer DJ (1991). Diet, behavior modification, and exercise: a review of obesity treatments from a long-term perspective. South Med J.

[31] Sjostrom L (2013). Review of the key results from the Swedish obese subjects (SOS) trial - a prospective controlled intervention study of bariatric surgery. J Intern Med.

[32] Flum DR, Belle SH, King WC, Wahed AS, Berk P, Longitudinal Assessment of Bariatric Surgery C (2009). Perioperative safety in the longitudinal assessment of bariatric surgery. N Engl J Med.

[33] Conaty EA, Denham W, Haggerty SP, Linn JG, Joehl RJ, Ujiki MB (2020). Primary care Physicians' perceptions of bariatric surgery and major barriers to referral. Obes Surg.

[34] Cardoso L, Rodrigues D, Gomes L, Carrilho F (2017). Short- and long-term mortality after bariatric surgery: a systematic review and meta-analysis. Diabetes Obes Metab.

[35] Carlsson LMS, Sjöholm K, Jacobson P, Andersson-Assarsson JC, Svensson PA, Taube M, Carlsson B, Peltonen M (2020). Life expectancy after bariatric surgery in the Swedish obese subjects study. N Engl J Med.

[36] Auspitz M, Cleghorn MC, Azin A, Sockalingam S, Quereshy FA, Okrainec A, Jackson TD (2016). Knowledge and perception of bariatric surgery among primary care physicians: a survey of family doctors in Ontario. Obes Surg.

[37] Stanford FC, Johnson ED, Claridy MD, Earle RL, Kaplan LM. The role of obesity training in medical school and residency on bariatric surgery knowledge in primary care physicians. Int J Fam Med. 2015:841249. 10.1155/2015/841249 PMC4539067 26339506

[38] Funk LM, Jolles SA, Voils CI (2016). Obesity as a disease: has the AMA resolution had an impact on how physicians view obesity?. Surg Obes Relat Dis.

[39] Glauser TA, Roepke N, Stevenin B, Dubois AM, Ahn SM (2015). Physician knowledge about and perceptions of obesity management. Obes Res Clin Pract.

[40] Salinas GD, Glauser TA, Williamson JC, Rao G, Abdolrasulnia M (2011). Primary care physician attitudes and practice patterns in the management of obese adults: results from a national survey. Postgrad Med.

[41] Rose PW, Rubin G, Perera-Salazar R, Almberg SS, Barisic A, Dawes M, Grunfeld E, Hart N, Neal RD, Pirotta M, Sisler J, Konrad G, Toftegaard BS, Thulesius H, Vedsted P, Young J, Hamilton W, The ICBP Module Working Group, Dawes D, Elwood M, Forsdike K, Hawryluk B, Knudsen AK, Lagerlund M, McAulay C, Mou J, Pirotta M, Sisler J, Weller D, The ICBP Module 3 Working Group* (2015). Explaining variation in cancer survival between 11 jurisdictions in the international Cancer benchmarking partnership: a primary care vignette survey. BMJ Open.

