# Supplementary material for: Primary care physicians’ knowledge, attitudes and concerns about bariatric surgery and the association with referral patterns: a Swedish survey study
Source: BMC Endocr Disord. 2021 Apr 8;21:62. doi: 10.1186/s12902-021-00723-8 (PMC8030650; doi:10.1186/s12902-021-00723-8)
Supplement: Supplementary file 1 — Additional file 1: Appendix 1. How we developed the indices [file 12902_2021_723_MOESM1_ESM.docx]

Appendix 1

How we developed the indices:

The index score for *referral pattern* comes from items 7-10, where the respondents could get 1-5 points on each item. High points were given if the PCP was more willing to refer. For item 9 we did a reverse calculation, i. e. low points were given if it was usually the patient that proposed a referral for bariatric surgery.

The index score for *knowledge* comes from items 23-41. The respondents could get one point for each correct answer. If the respondents had chosen alternative 4 or 5 for items 25-33, they received one point for each item. Item 41 contained several correct answers where the respondents could get 0,5 points for each correct answer (maximum 3 points). If the respondents gave the wrong answer or answered *do not know* they were given zero points for that specific item. The total amount of points was calculated to create the index score for knowledge.

The index score for *attitude* comes from items 47, 51 and 53. In item 53 the answer *very positive* was alternative 1 and *very negative* was alternative 5. Here we also did reverse calculations to fit with the answers from the other items. Respondents were given 1-5 points on each item depending on how they answered. The median score was 10 for the sum of these three items, and this score was given to respondents who had answered *do not know* for items 47, 51 and 53. We chose to give the points of the median score to these respondents because the answer *do not know* is not equal to not having any attitude at all. Very negative attitude was given low points, very positive attitude was given high points.

The index for *concern* comes from items 42-46. The respondents could get 1-5 points on each item depending on if they were minimally concerned or much concerned. Very much concern was given high points. Those who had given the answer *do not know* on any item was given the points of the median score which was 16. We chose to give a median score to these respondents considering that the answer “do not know” is not equivalent to not having any concerns. There was one respondent with missing answers for items regarding attitude and concern. This respondent was excluded from the analysis regarding attitude and concern and in the table 4 this respondent was given the value zero. For more information see table 3.
