# Supplementary material for: Primary care physicians’ knowledge, attitudes and concerns about bariatric surgery and the association with referral patterns: a Swedish survey study
Source: BMC Endocr Disord. 2021 Apr 8;21:62. doi: 10.1186/s12902-021-00723-8 (PMC8030650; doi:10.1186/s12902-021-00723-8)
Supplement: Supplementary file 2 — Additional file 2: Appendix 2. English translation of the questionnaire [file 12902_2021_723_MOESM2_ESM.docx]

Appendix 2

English translation of the questionnaire

Demographic information

1. You are:
   - - Male
     - Female
2. Your age:

- <35
- 35-49
- >49

1. You are:

- specialist in general practice
- Primary care physician without speciality in general practice

1. If you are specialist in family medicine, how many years have you worked as a specialist?

- 0-5 years
- 6-10 years
- 10 years

1. You work clinically

- Fulltime
- Part-time

1. You work at a

- Private primary health care
- Public primary health care

**To what extent do you agree or disagree with the following statements? (1= strongly disagree, 5=strongly agree)**

1. I refer patients for bariatric surgery if they fulfil the criteria for bariatric surgery.
2. 2) 3) 4) 5)
3. I often suggest referral for bariatric surgery to a patient, who meets criteria for bariatric surgery, even if I see the patient due to another illness.
4. 2) 3) 4) 5)
5. It is usually the patients themselves, who bring up the question of bariatric surgery.
6. 2) 3) 4) 5)
7. It is usually I that suggest a referral for bariatric surgery.
8. 2) 3) 4) 5)
9. Comments…..
10. When you suggest bariatric surgery to a patient, what is the main reason?

- Patient’s severe and complex obesity
- An already established or risk for comorbidities due to severe and complex obesity
- Difficulty to treat patient’s metabolic syndrome (e. g. diabetes type 2)
- Other reasons (please state under point 13)

1. Other ………
2. If a patient declines your suggestion about referral to bariatric surgery, what is the main reason?

- Risk for postoperative complications
- Long term consequences of the operation is not well known
- The results of the bariatric surgery on comorbidities are not well known
- Weight loss after bariatric surgery are not permanent
- The patient’s insufficient Swedish language ability
- The patient having difficulty to utilize substitution treatment postoperatively
- The patient would rather continue with lifestyle changes
- The patient having a bad attitude toward bariatric surgery due to an acquainted bad experience
- I do not know
- Other reasons (Please state under the point 15)

1. Other reasons…..
2. Has it mostly been male or female patients that you referred to bariatric surgery?

- Male
- Female
- No gender differences
- I do not know

1. In case you suggested bariatric surgery to a patient, has there been a gender difference in acceptability?

- Male patients accepted the referral to bariatric surgery more often than female patients.
- Female patients accepted the referral to bariatric surgery more often than male patients.
- There has been no gender difference
- I do not know

1. If there is a gender difference among patients who undergo bariatric surgery, what do you believe the reason could be? (multiple answers can be chosen)

- One sex requests bariatric surgery more often than the other
- Aesthetic aspects
- Psychological disease due to severe and complex obesity
- Cultural differences such as country of origin or religion
- Differences in diagnosis of obesity between the sexes (e. g. due to different body constitution)
- One sex cares more about one’s health
- Desire to have children
- Concern about the postoperative complications
- I do not know
- Other reasons (Please state under the point 19)

1. Other reasons…..
2. How many patients do you see per month, which are eligible for bariatric surgery?

- <1
- 1–2
- 3–5
- 6–10
- >10
- I do not know

1. How many patients have you referred to bariatric surgery in the LAST 5 YEARS?

- No one
- 1–2
- 3–5
- 6–10
- >10
- I do not know

**To what extent do you agree or disagree with the following statements?**

**(1= strongly disagree, 5=strongly agree)**

1. I have knowledge of the criteria concerning referral for bariatric surgery.
2. 2) 3) 4) 5)
3. Diet and exercise are effective methods for long-term weight loss in patients with severe and complex obesity.
4. 2) 3) 4) 5)
5. Bariatric surgery is the only effective long-term treatment for weight loss in patients with severe and complex obesity.
6. 2) 3) 4) 5)

**Do you believe that bariatric surgery can improve the following conditions associated with severe and complex obesity?** **(1= strongly disagree, 5=strongly agree)**

1. Diabetes mellitus type II 1) 2) 3) 4) 5) 6) I do not know
2. Hypertension 1) 2) 3) 4) 5) 6) I do not know
3. Hyperlipidaemia 1) 2) 3) 4) 5) 6) I do not know
4. Sleep apnea 1) 2) 3) 4) 5) 6) I do not know
5. Gastroesophageal reflux disease (GERD) 1) 2) 3) 4) 5) 6) I do not know
6. Pseudotumor cerebri 1) 2) 3) 4) 5) 6) I do not know
7. Polycystic ovary syndrome (PCOS) 1) 2) 3) 4) 5) 6) I do not know
8. Female infertility 1) 2) 3) 4) 5) 6) I do not know
9. Joint pain 1) 2) 3) 4) 5) 6) I do not know
10. How effective are medications/diets/exercise programs to achieve permanent weight loss in patients with severe and complex obesity?

- 10%
- 20%
- 40%
- 60%
- 80%
- I do not know

1. At what minimum BMI would you consider referring a patient WITHOUT comorbidities (hypertension, diabetes mellitus, sleep apnea) to undergo bariatric surgery?

- BMI 30
- BMI 35
- BMI 40
- BMI 45
- BMI >50
- I do not know

1. At what minimum BMI would you consider referring a patient WITH comorbidities (hypertension, diabetes mellitus, sleep apnea) to undergo bariatric surgery?

- BMI 30
- BMI 35
- BMI 40
- BMI 45
- BMI >50
- I do not know

1. Remission of diabetes is a known benefit of bariatric surgery. Approximately how long after surgery will patients see this benefit?

- Days after surgery
- 6 months after surgery
- Improvement of diabetes mirrors weight loss
- Improvement of diabetes occurs after significant weight loss has been achieved and maintained
- I do not know

1. Remission of type II diabetes mellitus occurs in what percentage of patients who have undergone bariatric surgery?

- 20%-40%
- 60%
- ≥80%
- I do not know

1. How long is a typical hospital stay for a patient undergoing laparoscopic bariatric surgery?

- 24–48 hours
- 3–5 days
- One week
- I do not know

1. What is the mortality rate associated with bariatric surgery?

- <1%
- 2%
- 6%
- >10%
- I do not know

1. Common postoperative side effects of bariatric surgery include (select all that apply):

- Alopecia (hair loss)
- Anaemia
- Fatigue
- Changes in taste sensation
- Irregular menstrual bleeding
- Bowel obstruction
- Nausea/vomiting
- Dumping
- I do not know

**To what extent do you agree or disagree with the following statements?**

**I am hesitant to refer patients for bariatric surgery because…**

**(1= strongly disagree, 5=strongly agree)**

1. I am concerned about the risk associated with the operation (e.g. anastomosis leakage, infection, bleeding)

1) 2) 3) 4) 5) 6) I do not know

1. I am concerned about postoperative surgical complications (e.g. gastric ulcer, internal hernia, gallstones and chronic abdominal pain)

1) 2) 3) 4) 5) 6) I do not know

1. I am concerned about postoperative medical complications (e.g. mineral and vitamin deficiencies, hypoglycaemia, dumping)

1) 2) 3) 4) 5) 6) I do not know

1. I am concerned about the risk of psychiatric side effects following the bariatric surgery (e.g. depression, drug abuse)

1) 2) 3) 4) 5) 6) I do not know

1. Lack of long-term data on the risk and side effects and complications of bariatric surgery

1) 2) 3) 4) 5) 6) I do not know

1. I have bad experience of bariatric surgery.

1) 2) 3) 4) 5) 6) I do not know

1. I am content to prescribe non-surgical intervention for weight management to my patients with obesity

1) 2) 3) 4) 5) 6) I do not know

**To what extent do you agree or disagree with the following statements?**

**(1= strongly disagree, 5=strongly agree)**

1. I feel qualified to discuss bariatric surgery as a treatment option with my patients who have severe and complex obesity.

1) 2) 3) 4) 5) 6) I do not know

1. I feel qualified to take care of my patients after bariatric surgery.

1) 2) 3) 4) 5) 6) I do not know

1. I believe that the benefits of bariatric surgery are worth the risks of the operation.

1) 2) 3) 4) 5) 6) I do not know

1. I need more information about the different aspects of gastric bypass surgery.

1) 2) 3) 4) 5) 6) I do not know

1. What is your overall impression of bariatric surgery as a treatment for obesity related comorbidities?

- very positive
- positive
- neutral
- negative
- very negative

1. Have you ever treated patients who have undergone bariatric surgery?

- yes
- No

1. Have you ever treated patients with complications after bariatric surgery?

- Yes
- No

1. Have you ever refused to refer a patient to bariatric surgery despite the patient’s request?

- Yes
- No
- I do not know

1. What has been your reason for refusing to refer patients to bariatric surgery? (select all that apply)

- I have never refused to refer a patient to bariatric surgery.
- Risk for postoperative complications
- The long-term consequences of bariatric surgery are not completely known.
- Weight loss after bariatric surgery is not permanent.
- The patient was not medically eligible.
- It was unclear if the patient could follow the postoperative advices/restrictions.
- The patient had cognitive impediment.
- The patient had difficulty understanding Swedish language.
- The costs for bariatric surgery is a burden for society and healthcare system.
- Other reasons (please state under the point 58)

1. Other reasons…..
2. Please share your perspective on bariatric surgery for the treatment of patients with chronic obesity or give further comments……

Thank you for your participation
